# Peripheral Arterial Disease prevalence and risk factors in the Eastern Caribbean Health Outcomes Research Network (ECHORN) cohort

**DOI:** 10.1371/journal.pone.0306918

**Published:** 2024-08-26

**Authors:** O. Peter Adams, Deron Galusha, Josefa L. Martinez-Brockman, Euclid H. Morris, Saria Hassan, Rohan G. Maharaj, Cruz M. Nazario, Maxine Nunez, Marcella Nunez-Smith

**Affiliations:** 1 Department of Clinical Sciences, Faculty of Medical Sciences, The University of the West Indies, Cave Hill Campus, Barbados; 2 Equity Research and Innovation Center, Yale School of Medicine, New Haven, Connecticut, United States of America; 3 Department of Internal Medicine, Yale School of Medicine, New Haven, Connecticut, United States of America; 4 Emory University School of Medicine, Atlanta, Georgia, United States of America; 5 Department of Paraclinical Sciences, University of the West Indies, Saint Augustine, Trinidad and Tobago; 6 Department of Biostatistics and Epidemiology, Graduate School of Public Health, University of Puerto Rico at Medical Sciences Campus, San Juan, Puerto Rico; 7 School of Nursing, University of the Virgin Islands, St. Thomas, Virgin Islands, United States of America; Atal Bihari Vajpayee Indian Institute of Information Technology and Management, INDIA

## Abstract

**Introduction:**

Peripheral arterial disease (PAD) indicates generalised atherosclerotic disease but is often asymptomatic. The prevalence and potential risk factors of PAD were studied in ECHORN cohort study participants.

**Methods:**

Representative samples of community-dwelling people ≥40 years of age residing in Barbados, Puerto Rico, Trinidad, and the USVI were recruited. The survey included questions on diabetes, hypertension, heart disease and smoking status. Body Mass Index, HbA1c, blood glucose and lipids were determined. Ankle brachial index (ABI) was evaluated in one leg. An oscillometric device measured arm and leg systolic BP simultaneously. ABI classifications were PAD ≤0.90, borderline 0.91 to 0.99, normal 1.00 to 1.40, and non-compressible >0.40. Multivariable logistic regression tested associations of potential risk factors with PAD.

**Results:**

Of 2772 participants (mean age 57.3, 65.2% female), 35.8% were overweight, 38.1% obese, 32.4% had diabetes, 60% hypertension, and 15.4% reported heart. ABI prevalence (95% CI) by category was PAD 4.4% (3.6%, 5.1%), borderline 5.2% (4.4%, 6.1%), normal 87.0% (85.8%, 88.3%) and noncompressible 3.4% (2.7%, 4.0%). Female sex (OR 1.72, 95% CI 1.07 to 2.77), diabetes (OR 2.23, 95% CI 1.47 to 3.4), heart disease history (OR 1.74, 95% CI 1.07 to 2.83) and less than high school education vs having a university degree (OR 2.49, 95% CI 1.19 to 5.22) were independently associated with PAD.

**Conclusions:**

Testing one leg only would underestimate PAD prevalence. Increasing the ABI cutoff for identifying PAD to <1.0 when using oscillometric devices is suggested by some studies but would more than double the estimated prevalence. Guidelines need to address this issue. Female sex and lower educational attainment are important considerations when screening. While diabetes and a history of heart disease were confirmed as risk factors, the lack of association of increasing age and cigarette smoking with PAD was unexpected.

## Introduction

Peripheral arterial disease (PAD), the partial or complete obstruction of peripheral arteries, is a manifestation of systemic atherosclerosis and a marker for future cardiovascular events, morbidity and mortality [1]. There is an associated 3 to 6 fold increased risk of death from cardiovascular causes [2, 3], and PAD is classified as a coronary heart disease risk equivalent [4]. PAD is a useful indicator for the secondary prevention of cardiovascular disease only if the condition is identified. It is often asymptomatic, and in the USA primary care physician and public awareness is low [5, 6]. Awareness is also likely to be low in the Eastern Caribbean islands of Barbados and Trinidad and Tobago where few people with diabetes have an adequate foot examination in primary care [7, 8].

PAD is estimated to have a prevalence of 7.4% in high-income countries (HICs) and 5.1% in low and middle income countries (LMICs) in people older than 25 years. There is a sharp increase in prevalence with increasing age although the increase is less marked in LMICs compared to HICs [9]. Other associated factors include cigarette smoking, hyperlipidaemia, hypertension [9–11] and a family history of PAD [12, 13]. In the USA the prevalence in those 40 years and older is 4.3%, and in age and sex adjusted logistic regression, non- Hispanic black race or ethnicity, current smoking, diabetes, hypertension, hypercholesterolemia and low kidney function were positively associated with PAD [1]. Other studies have shown that the prevalence of PAD is higher in people of African descent compared to non-Hispanic whites and this is not explained by higher levels of diabetes, hypertension and obesity [10, 14].

Digital subtraction angiography and CT angiography are considered gold standards for diagnosing peripheral arterial disease [13, 15, 16]. Both tests are expensive and invasive. It is recommended that the resting ankle brachial pressure index (ABI), be used to establish the diagnosis of PAD [13] and this is the accepted method used in epidemiological studies [9]. An ABI ≤0.90 is diagnostic of PAD and >1.40 indicates noncompressible arteries which may mask PAD and is more common in people with diabetes and chronic kidney disease [13]. Determining the ABI by measuring arm and ankle systolic pressures with a Doppler device is considered the non-invasive gold standard but requires trained personnel and is time consuming [17, 18]. Compared to angiography it has a 90% sensitivity and 98% specificity for detecting a ≥50% stenosis [5]. Oscillometric devices allow automated and simultaneous arm and leg systolic BP measurements, and reduce training requirements, time, and inter and intra observer variability and biases associated with the use of Doppler ultrasound [19, 20]. A meta-analysis estimated a 65% sensitivity and 96% specificity compared to Doppler measurement [17]. Other primary studies done since that meta-analysis have found sensitivities of around 90% [20, 21]. Blood Pressure (BP) taken above the ankle with an oscillometric device may be higher than those measured in the foot arteries with a Doppler device [22]. It has been suggested that a higher ABI threshold of 1.0 for diagnosing PAD may be preferable to increase the sensitivity when an oscillometric method is used [21, 23]. Compared to Doppler ultrasound the sensitivity increased from 86 to 94% by using a threshold of 1.0 rather than 0.9 with oscillometric devices while the specificity fell from 95 to 92% [21]. In a direct comparison to CT angiography as the gold standard, in a population with a 78% prevalence of >50% stenosois, the optimal ABI cutoff to detect this level of stenosis using an oscillometric device was 0.99 and this resulted in 90% sensitivity and 85% specificity [15]. In another study with a 7% prevalence of ≥50% stenosis determined by digital subtraction angiography as the gold standard, the optimal cutoff was an ABI of 0.95 which resulted in a 91% sensitivity and 86% specificity [16]. Better agreement between Doppler and oscillometric devices has also been shown when a per subject classification (PAD in either leg) was done as opposed to a per leg analysis [17]. Further, multi-cuff oscillometric devices designed to measure ABI with simultaneous arm and leg measurements were found to perform better than single cuff devices [20].

The ECHORN cohort has empanelled a sample of the non-institutionalised individuals 40 years of age and over, on Barbados, Puerto Rico, Trinidad and the United States Virgin Islands (USVI). These islands are all classified as having high-income economies by the World Bank [24]. The populations have a high prevalence of diabetes and hypertension, both risk factors for PAD. In Barbados and Trinidad and Tobago cardiovascular diseases account for 30% of all deaths [25] while in Puerto Rico and the USVI, cardiovascular diseases account for 18.4% and 26.8% of all deaths, respectively [26]. There have been few studies on PAD prevalence in the region. In Barbados the prevalence of PAD was estimated to be 18.6% in a population-based sample of people with known and newly identified diabetes [27]. This population also had a high prevalence of peripheral neuropathy [28]. The objectives of our study were to (1) estimate the prevalence of PAD among ECHORN cohort participants and (2) identify potential risk factors associated with PAD.

## Materials and methods

Baseline data from the ECHORN Cohort Study were used. ECHORN is a population-based longitudinal study of community-dwelling adults 40 years and older in Barbados, Puerto Rico, Trinidad, and the USVI.

Stratified multi-stage probability sampling was used on the islands of Puerto Rico, Barbados, and Trinidad. In Barbados, participants were recruited from households within randomly selected enumeration districts across the entire island. In the larger islands of Puerto Rico and Trinidad adults were recruited from households from randomly selected districts within two regions. Simple random sampling was used on the USVI where adults across the entire territory were invited to participate through random digit dialling. Research activities occurred at community assessment centres staffed by a research team on each island. Between 2013 and 2018, 2,961 persons were recruited to the cohort. A complete accounting of the baseline methodology is published elsewhere [29].

Participants completed a baseline survey which included questions on diabetes and hypertension diagnosis. Survey items were self-administered on a tablet computer. This was assisted where required by computer generated audio or with the help of a research assistant. The physical examination included measurement of weight and height which were used to calculate Body Mass Index (BMI). BMI categories were underweight <18 Kg/m^2^, normal weight 18 to 24.9 Kg/m^2^, overweight 25 to 29.9 Kg/m^2^, obese 30 to 34.9 kg/m^2^ and obese class II and III ≥35 Kg/m^2^ [30]. Waist and hip circumferences were measured, and the waist hip ratio calculated. Ratios >0.9 for men, >0.85 for women were considered elevated [31]. Blood was drawn for HbA1c, blood glucose and lipid estimation. Participants were classified as having diabetes from a self-report of a health care worker diagnosis and/or taking medication for diabetes and/or from laboratory testing (HbA1c ≥6.5% and/or fasting plasma glucose ≥126 mg/dL). Hypertension status was determined by a report of taking medication for hypertension and/or an average of three BP readings taken one minute apart ≥140/90. The arm with the higher average systolic BP was used. Education level was classified as not completing high school, completing high school, some college which could include having an associate degree, and college + which meant having a university degree. Heart disease was defined as a self-report one or more of the following conditions: coronary heart disease, angina pectoris, abnormal heart rhythm, heart attack, or congestive heart failure. Physical activity was estimated by the WHO Global Physical Activity Questionnaire and classified as low, moderate and high [32]. Current smoking was defined as smoking at least 20 cigarettes or 1 cigar or half an ounce sachet of loose tobacco per month. ASCVD 10-year risk was calculated according to the American College of Cardiology/American Hypertension Association guidelines [33].

A WatchBP^®^ (Microlife Switzerland) office automated ABI machine was used to determine both BP and ABI. The BP of both arms was first measured simultaneously after the participant was seated for 5 minutes, back supported, feet on the ground and the cuffs at heart level. The machine took 3 readings automatically, one minute apart and then averaged them. The arm with the higher average systolic pressure and ankle on the same side were then used to determine the ABI after the participant had been quietly lying supine for at least 5 minutes. The lower edge of the ankle cuff was placed 2 to 3 cm above the ankle with the artery mark over the posterior tibial artery. The lower edge of the arm cuff was placed 2 to 3 cm above the antecubital fossa with the artery mark over the brachial artery. The arm and ankle cuffs measured ankle and brachial systolic pressures simultaneously and the machine calculated the ABI. For each participant only one leg was assessed.

### Analysis

Data were analysed using SAS version 9.4 (SAS Institute Inc., Cary, North Carolina). ABI values were categorised into PAD (ABI ≤0.90), borderline (ABI 0.91.-0.99), normal (ABI 1.00 to 1.40) and noncompressible (ABI >1.40) [13]. Prevalence and 95% confidence interval of each ABI category by characteristics hypothesised to be risk factors for PAD, including age, sex, chronic disease diagnosis and other cardiovascular risk factors were first explored. Differences between PAD and normal ABI groups are presented with 95% confidence intervals.

Unadjusted odds ratios and *p* values for people with PAD compared to those with normal ABIs were first determined. Multivariable logistic regression analysis with backward elimination was conducted using all associations involving PAD with a *p* value <0.2 to create a multivariable model containing only variables with a *p* value <0.05. Age, sex, education level, diabetes, hypertension, heart condition, history of smoking, treated hypertension, systolic and diastolic blood pressure groups, BMI groups, and physical activity groups were entered into the backward elimination model as independent variables predicting the presence of PAD as the dependent variable. An additional model was analyzed using systolic and diastolic blood pressure, and BMI values as continuous independent variables.

To account for the possibility that the ABI threshold for PAD should be <1.00 when using an oscillometric device [15, 21, 23], PAD was also defined as an ABI <1.00 and compared to people with normal ABIs (1.00 to 1.40). Multivariable logistic regression was conducted as above on variables with a *p* value <0.2. Age, sex, education level, diabetes, hypertension, history of smoking, treated hypertension, systolic and diastolic blood pressure groups, BMI groups, and physical activity groups were entered into the backward elimination model as independent variables. An additional model was analyzed using systolic and diastolic blood pressure, and BMI values as continuous independent variables.

As noncompressible arteries may mask PAD, this category (ABI >1.4) was combined with PAD (ABI <0.9) and a similar analysis done.

### Ethical approval

The ECHORN Cohort Study received approval from the Yale University Human Subjects Investigation Committee and the Institutional Review Boards of the University of Puerto Rico Medical Sciences Campus, the University of the US Virgin Islands, the University of the West Indies Cave Hill Campus, the University of the West Indies St. Augustine campus, and the Ministry of Health of Trinidad and Tobago. Informed consent was obtained from all participants prior to enrollment.

## Results

**Fig 1:**
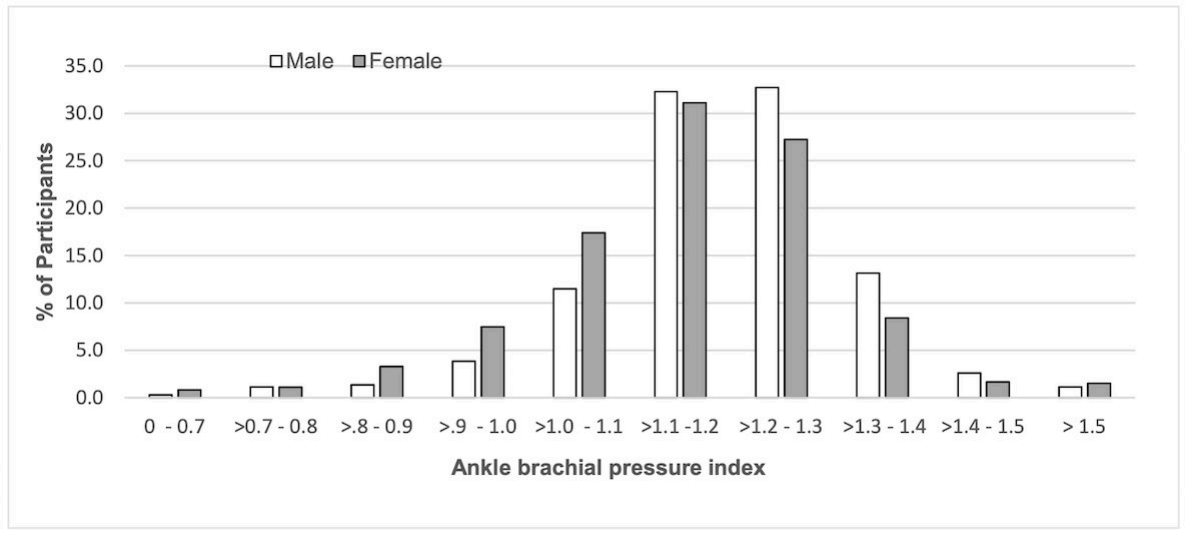
The distribution of the ankle brachial pressure index (ABI) by sex.

Of the 2961 people recruited to the cohort, the 2772 with a valid ABI measurement had a mean ABI of 1.17 (range 0.39 to 2.58), mean age of 57.3 years (SD 10.2) and 65.2% were female. Fig 1 shows the ABI distribution by sex. One quarter (24.9%) were normal weight, 35.8% overweight, 38.1% obese, 50.2% had an increased waist/hip ratio, 32.4% had diabetes; 60.0% hypertension, 15.4% reported a heart condition and 3.5% a history of stroke. Of the 2772 people, 996 were from Barbados (mean age 57.5, 70% female), 818 from Trinidad and Tobago (mean age 56.3, 61% female), 744 from Puerto Rico (mean age 58.1, 66% female), and 214 from USVI (mean age 56.8, 58% female).

The prevalence of PAD (ABI ≤0.9) in the assessed leg was 4.4% (95% CI 3.6% to 5.1%) with 121 out of 2772 participants having PAD in the leg that was evaluated. PAD prevalence was 2.8% (95% CI 1.8% to 3.8%) for males and 5.2% (95% CI 4.2% to 6.2%) for females (*p* = 0.0031). For those with diabetes the PAD prevalence was 6.6% (95% CI 4.9% to 8.4%) vs 3.1% (95% CI 2.2% to 3.9%) for those without (*p* = < 0.0001). Cohort members from Barbados were most likely to have PAD (6.7% [95% CI 5.2 to 8.3]) and those from Puerto Rico least likely (2.4% [95% CI 1.3 to 3.5]) (Table 1).

**Table 1:** Prevalence of ankle brachial pressure category by various characteristics in adults 40 years of age an older in the ECHORN cohort study.

Characteristic	Total (n = 2,772)	PAD (n = 121)	Borderline PAD (n = 145)	Normal (n = 2,413)	Non-compressible (n = 93)
		(ABI ≤0.9)	(ABI .91–.99)	(ABI 1.0–1.4)	(ABI >1.40)
		% (95% CI)	% (95% CI)	% (95% CI)	% (95% CI)
**Total**	2772	4.4 (3.6–5.1)	5.2 (4.4–6.1)	87.0 (85.8–88.3)	3.4 (2.7–4.0)
**Sex**					
Male	966	2.8 (1.8–3.8)	3.1 (2–4.2)	90.4 (88.5–92.2)	3.7 (2.5–4.9)
Female	1806	5.2 (4.2–6.2)	6.4 (5.2–7.5)	85.3 (83.6–86.9)	3.2 (2.3–4.0)
**Age group years**					
40–49	693	4.3 (2.8–5.8)	4.9 (3.3–6.5)	87.7 (85.3–90.2)	3.0 (1.8–4.3)
50–59	988	3.8 (2.6–5)	4.4 (3.1–5.6)	87.8 (85.7–89.8)	4.0 (2.8–5.3)
60–69	719	4.0 (2.6–5.5)	5.7 (4.0–7.4)	87.1 (84.6–89.5)	3.2 (1.9–4.5)
70+	372	6.5 (4–8.9)	7.3 (4.6–9.9)	83.9 (80.1–87.6)	2.4 (0.9–4)
**Education level**					
Not completed High School	957	5.2 (3.8–6.6)	6.2 (4.6–7.7)	85.9 (83.7–88.1)	2.7 (1.7–3.7)
Completed High School	629	5.6 (3.8–7.4)	4.9 (3.2–6.6)	86.6 (84–89.3)	2.9 (1.6–4.2)
Some college	578	4.2 (2.5–5.8)	5.7 (3.8–7.6)	86.0 (83.2–88.8)	4.2 (2.5–5.8)
University degree	479	1.9 (0.7–3.1)	4.2 (2.4–6.0)	89.6 (86.8–92.3)	4.4 (2.6–6.2)
**ECHORN site**					
Barbados	996	6.7 (5.2–8.3)	5.8 (4.4–7.3)	85 (82.8–87.3)	2.4 (1.5–3.4)
Puerto Rico	744	2.4 (1.3–3.5)	4.3 (2.8–5.8)	87.6 (85.3–90)	5.6 (4.0–7.3)
Trinidad	818	3.5 (2.3–4.8)	5.6 (4.0–7.2)	88.8 (86.6–90.9)	2.1 (1.1–3.1)
USVI	214	3.3 (0.9–5.7)	4.2 (1.5–6.9)	87.9 (83.5–92.2)	4.7 (1.8–7.5)
**Diabetes** ^a^					
No	1602	3.1 (2.2–3.9)	4.3 (3.3–5.3)	89.3 (87.8–90.8)	3.3 (2.4–4.2)
Yes	767	6.6 (4.9–8.4)	6.9 (5.1–8.7)	82.4 (79.7–85.1)	4.0 (2.6–5.4)
**Hypertension** ^b^					
No	1105	3.3 (2.3–4.4)	5 (3.7–6.3)	88.3 (86.4–90.2)	3.3 (2.3–4.4)
Yes	1658	5.1 (4–6.1)	5.4 (4.3–6.5)	86.2 (84.5–87.8)	3.4 (2.5–4.2)
**Heart condition** ^c^					
**No**	2332	3.8 (3–4.6)	5.2 (4.3–6.1)	87.8 (86.5–89.1)	3.2 (2.5–3.9)
**Yes**	424	7.1 (4.6–9.5)	5.2 (3.1–7.3)	83.5 (80.0–87.0)	4.2 (2.3–6.2)
**History of stroke**					
No	2668	4.3 (3.5–5)	5.1 (4.3–6.0)	87.3 (86–88.6)	3.3 (2.6–4)
Yes	96	7.3 (2.1–12.5)	7.3 (2.1–12.5)	80.2 (72.2–88.2)	5.2 (0.8–9.7)
**Treated Hypertension**					
No	1637	3.9 (3–4.8)	4.7 (3.7–5.7)	87.9 (86.3–89.5)	3.5 (2.6–4.4)
Yes	1129	5 (3.8–6.3)	5.9 (4.6–7.3)	85.8 (83.8–87.9)	3.2 (2.2–4.2)
**Diastolic BP mmHg**					
<80	1333	3.8 (2.7–4.8)	5.6 (4.3–6.8)	86.6 (84.8–88.5)	4.1 (3.0–5.1)
80–89	883	4.8 (3.4–6.2)	5.3 (3.8–6.8)	86.9 (84.6–89.1)	3.1 (1.9–4.2)
90–99	399	4.8 (2.7–6.9)	3.8 (1.9–5.6)	89.0 (85.9–92)	2.5 (1.0–4.0)
100+	146	6.8 (2.8–10.9)	6.2 (2.3–10.1)	85.6 (79.9–91.3)	1.4 (-0.5–3.3)
**Systolic BP mmHg**					
<120	498	3.6 (2–5.3)	5.2 (3.3–7.2)	87.3 (84.4–90.3)	3.8 (2.1–5.5)
120–129	595	3 (1.6–4.4)	4.5 (2.9–6.2)	88.2 (85.6–90.8)	4.2 (2.6–5.8)
130–139	617	3.9 (2.4–5.4)	3.9 (2.4–5.4)	89.1 (86.7–91.6)	3.1 (1.7–4.4)
140–149	446	7.0 (4.6–9.3)	4.5 (2.6–6.4)	84.5 (81.2–87.9)	4 (2.2–5.9)
150–159	200	3.0 (0.6–5.4)	5.0 (2–8)	89.5 (85.3–93.7)	2.5 (0.3–4.7)
160+	407	5.9 (3.6–8.2)	9.3 (6.5–12.2)	83 (79.4–86.7)	1.7 (0.5–3.0)
**BMI class Kg/m** ^**2**^					
Underweight (<18)	36	0.0 (0.0–0.0)	8.3 (0.0–17.4)	91.7 (82.6–100.7)	0.0 (0.0–0.0)
normal (18–24.9)	685	5.0 (3.3–6.6)	5.0 (3.3–6.6)	88.5 (86.1–90.9)	1.6 (0.7–2.5)
overweight (25–29.9)	986	2.9 (1.9–4.0)	4.6 (3.3–5.9)	89.9 (88.0–91.7)	2.6 (1.6–3.6)
obese (30–34.9)	616	5.0 (3.3–6.8)	6.3 (4.4–8.3)	84.3 (81.4–87.1)	4.4 (2.8–6.0)
obese class II+III (≥35)	433	5.8 (3.6–8.0)	5.3 (3.2–7.4)	82.2 (78.6–85.8)	6.7 (4.3–9.1)
**Waist/hip ratio** ^d^					
Normal	1374	4.1 (3.0–5.1)	5.2 (4.0–6.3)	87.7 (86–89.4)	3.1 (2.1–4)
Increased	1387	4.6 (3.5–5.7)	5.3 (4.2–6.5)	86.4 (84.6–88.2)	3.7 (2.7–4.7)
**Current smoking**					
No	2438	4.5 (3.7–5.3)	5.2 (4.3–6.0)	86.9 (85.5–88.2)	3.4 (2.7–4.2)
Yes	220	4.1 (1.5–6.7)	6.4 (3.1–9.6)	86.4 (81.8–90.9)	3.2 (0.9–5.5)
**Physical activity**					
Low	1141	4.8 (3.6–6.1)	6.6 (5.1–8.0)	84.8 (82.8–86.9)	3.8 (2.7–4.9)
Moderate	485	4.9 (3.0–6.9)	4.1 (2.4–5.9)	88.7 (85.8–91.5)	2.3 (0.9–3.6)
High	869	3.1 (2.0–4.3)	4.7 (3.3–6.1)	88.8 (86.7–90.9)	3.3 (2.1–4.5)
**Total cholesterol mg/dL**					
<200	1334	3.6 (2.6–4.6)	4.2 (3.1–5.3)	87.9 (86.2–89.7)	4.3 (3.2–5.4)
200–239	679	4.0 (2.5–5.4)	5.7 (4–7.5)	87.8 (85.3–90.2)	2.5 (1.3–3.7)
240+	259	5.0 (2.4–7.7)	6.6 (3.5–9.6)	86.5 (82.3–90.7)	1.9 (0.3–3.6)
**HDL mg/dL**					
<40	393	3.3 (1.5–5.1)	5.9 (3.5–8.2)	85.5 (82–89)	5.3 (3.1–7.6)
40–59	1204	3.9 (2.8–5)	4.7 (3.5–5.8)	88.0 (86.2–89.9)	3.4 (2.4–4.4)
60+	68	4.2 (2.6–5.7)	5.1 (3.4–6.8)	88.3 (85.8–90.7)	2.5 (1.3–3.7)
**LDL mg/dL**					
< 100	776	3.9 (2.5–5.2)	3.9 (2.5–5.2)	88.3 (86–90.5)	4.0 (2.6–5.4)
100–129	782	3.3 (2.1–4.6)	5.2 (3.7–6.8)	88 (85.7–90.3)	3.5 (2.2–4.7)
130+	695	4.6 (3.0–6.2)	6 (4.3–7.8)	86.3 (83.8–88.9)	3.0 (1.7–4.3)
**Triglycerides mg/dL**					
<150	1417	4.0 (2.9–5)	4.9 (3.8–6.1)	87.7 (85.9–89.4)	3.5 (2.5–4.4)
150–199	320	3.4 (1.4–5.4)	3.4 (1.4–5.4)	89.4 (86.0–92.8)	3.8 (1.7–5.8)
200+	349	3.4 (1.5–5.4)	5.4 (3.1–7.8)	86.8 (83.3–90.4)	4.3 (2.2–6.4)
**ASCVD 10-year risk**					
< = 7.5	1125	3.2 (2.2–4.2)	4.4 (3.2–5.6)	88.7 (86.9–90.6)	3.6 (2.5–4.7)
7.6–9.9	173	4.6 (1.5–7.8)	3.5 (0.7–6.2)	86.7 (81.6–91.8)	5.2 (1.9–8.5)
10–19.9	371	3.8 (1.8–5.7)	4.9 (2.7–7)	88.1 (84.9–91.4)	3.2 (1.4–5)
20+	263	6.5 (3.5–9.4)	7.2 (4.1–10.4)	82.1 (77.5–86.8)	4.2 (1.8–6.6)

^a^ Diabetes is defined as self-report, Hba1c ≥6.5% or fasting glucose ≥126 mg/dL, or self-reported medication use

^b^ Hypertension is defined as blood pressure ≥140 mm Hg systolic or ≥90 mm Hg diastolic, or self-reported medication use

^c^ Heart Condition is defined as self-reported coronary heart disease, angina, abnormal heart rhythm, heart attack, congestive heart failure or other heart condition

^d^ An elevated waist-to-hip-ratio is defined as >0.9 for men, >0.85 for women

**Fig 2:**
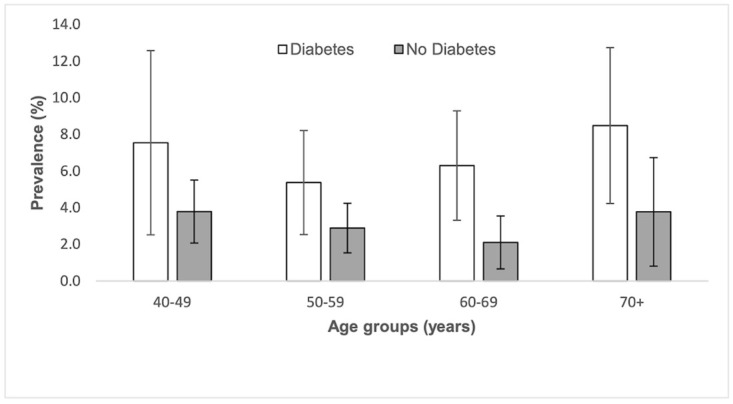
Peripheral arterial disease (ankle brachial pressure index ≤0.9) prevalence by age group and diabetes status^a^. Error bars represent 95% confidence intervals. ^a^ Diabetes is defined as self-report, Hba1c ≥6.5 or fasting glucose ≥126 mg/dL or self-reported medication use.

**Fig 3:**
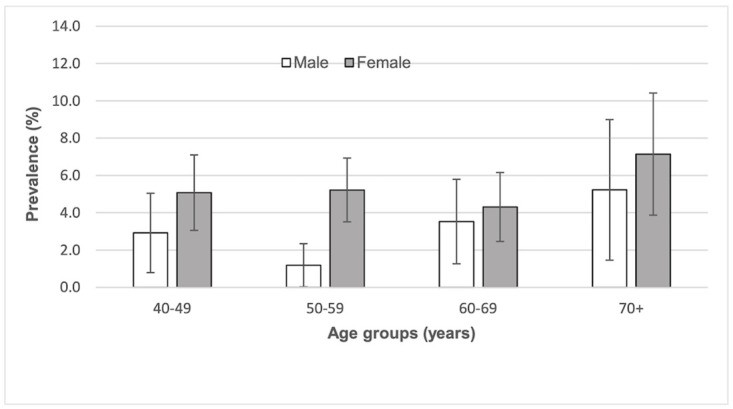
Prevalence of peripheral arterial disease (ankle brachial pressure index ≤0.9) by age group and sex. Error bars represent 95% confidence intervals.

**Fig 4:**
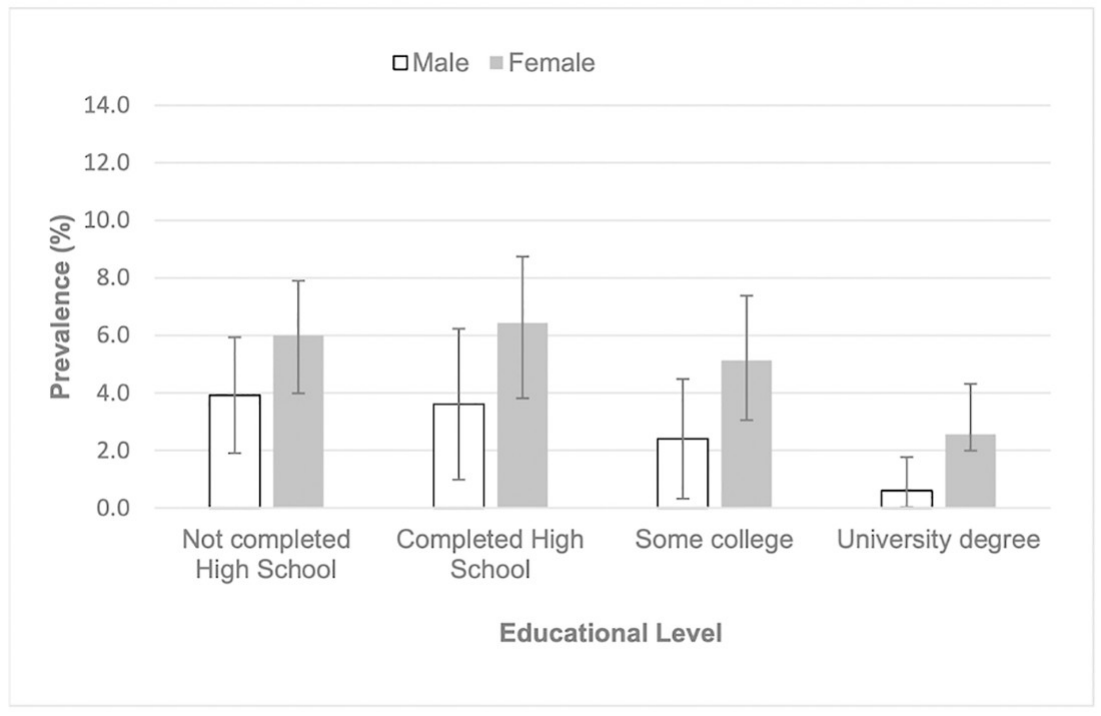
Prevalence of peripheral arterial disease (ankle brachial pressure index ≤0.9) by highest educational level attained and sex. Error bars represent 95% confidence intervals.

Women were more likely to have PAD than men with 77.7% of those having PAD being female vs 63.8% of those with a normal ABI (difference 13.9, 95% CI 5.1 to 22.6). Fifty one percent of people with PAD had diabetes compared to only 30.6% of those with a normal ABI (difference 20.4%, 95% CI 6.2 to 34.6). Fig 2 shows the distribution of PAD by age and diabetes. The mean age of those with PAD was slightly greater than those without PAD (difference 1.3 years, 95% CI 0.6 to 3.1) (Table 2) with those 70 years of age and over having the highest prevalence (Fig 3). PAD prevalence decreased with increasing education level (Fig 4).

**Table 2:** Comparison of risk factors of those with an ankle brachial pressure index (ABI) in the normal range (1.0 to 1.4) with those with Peripheral Arterial Disease (PAD) (ABI ≤0.9). Figures are means (SD) unless otherwise stated.

Risk Factor	Ankle Brachial Pressure Index			
% unless otherwise stated	PAD (ABI ≤0.9)	Normal (ABI 1.0–1.4)	Difference	(95% CIs)
Female sex (%)	77.7	63.8	13.9	(6.2, 21.5)
Age (years)	58.4 (SD 11.8)	57.1 (SD 10.2)	1.3	(0.6, 3.1)
Education level (%)				
Not completed High School	42.4	35.9	6.5	(-2.6, 15.6)
Completed High School	29.7	23.8	5.9	(-2.5, 14.3)
Some college	20.3	21.7	-1.4	(-8.8, 6.1)
University degree	7.6	18.7	-11.1	(-16.1, -6.0)
Diabetes^a^ (%)	51.0	30.6	20.4	(10.4, 30.4)
Hypertension^b^ (%)	69.4	59.4	10	(1.6, 18.4)
Treated Hypertension (%)	47.1	40.2	6.9	(-2.2, 16.0)
Heart condition^c^ (%)	25.2	14.7	10.5	(2.5, 18.4)
History of stroke (%)	5.8	3.2	2.6	(-1.6, 16.8)
Current smoking (%)	7.6	8.2	-0.6	(-5.6, 4.2)
Physical activity (%)				
Low	51.9	44.6	7.3	(-2.5, 17.0)
Moderate	22.6	19.8	2.8	(-5.3, 11.0)
High	25.5	35.6	-10.1	(-18.6, -1.6)
BMI (Kg/m^2^)	30 (SD 7.2)	28.9 (SD 5.8)	1.1	(0.0, 2.2)
Elevated waist/hip ratio^d^ (%)	53.3	49.9	3.4	(-5.7, 12.6)
Systolic BP (mmHg)	141.1 (SD 21.9)	136.9 (SD 20.5)	4.2	(0.5, 8)
HDL (mg/dL)	52.9 (SD 15.1)	52.7 (SD 14.7)	0.2	(-2.9, 3.4)
LDL (mg/dL)	116.6 (SD 39.1)	114.3 (SD 34.4)	2.2	(-5.2, 9.6)
Triglycerides (mg/dL)	129.3 (SD 82.5)	137.1 (SD 80.6)	-7.8	(-26, 10.4)

^a^ Diabetes is defined as self-report, Hba1c ≥6.5% or fasting glucose ≥126 mg/dL, or self-reported medication use)

^b^ Hypertension is defined as blood pressure ≥140 mm Hg systolic or ≥90 mm Hg diastolic, or self-reported medication use

^c^ Heart Condition is defined as self-reported coronary heart disease, angina, abnormal heart rhythm, heart attack, congestive heart failure or other heart condition

^d^ An elevated waist-to-hip-ratio is defined as >0.9 for men, >0.85 for women

After entering potential risk factors (age, sex, education level, diabetes, hypertension, heart condition, history of smoking, treated hypertension, systolic and diastolic blood pressure groups, BMI groups, and physical activity) of PAD (ABI ≤0.90) with a p-value ≤0.20 into a multivariable logistic regression we found that independent predictors for PAD were female sex (OR 1.72, 95% CI 1.07 to 2.77), diabetes (OR 2.23, 95% CI 1.47 to 3.40), a history of heart disease (OR 1.74, 95% CI 1.07 to 2.83) and less than high school education vs having a university degree (OR 2.49, 95% CI 1.19 to 5.22) and high school education vs having a university degree (OR 2.41, 95% CI 1.10 to 5.28) (Table 3). The independent predictors did not change when systolic and diastolic blood pressures, and BMI were entered as continuous variables in the model. If site was added to the model (Puerto Rico as the reference) then Barbados became an additional predictor (OR 2.31, 95% CI 1.31 to 4.06) and educational level was no longer significant.

**Table 3:** Unadjusted^a^ and adjusted odds ratios for predictors of Peripheral Arterial Disease (ABI ≤0.90) compared to normal arteries (ABI 1.0 to 1.4).

Factor	Unadjusted Odds ratio (95% CI)	*P* value	Adjusted Odds ratio (95% CI)	*P* value
Female	1.97 (1.28 to 3.05)	0.002	1.72 (1.07 to 2.77)	0.025
**Education Level**				
Not completed High School	2.90 (1.41 to 5.95)	0.004	2.49 (1.19 to 5.22)	0.016
Completed High School	3.06 (1.46 to 6.43)	0.003	2.41 (1.10 to 5.28)	0.028
Some college	2.30 (1.06 to 5.00))	0.036	2.14 (0.97 to 4.72)	0.061
University Degree	Reference		Reference	
Diabetes^b^	2.36 (1.58 to 3.53)	<0.001	2.23 (1.47 to 3.40)	<0.001
Heart Condition^c^	1.95 (1.27 to 3.00)	0.002	1.74 (1.07 to 2.83)	0.025

^a^ Age, sex, education level, diabetes, hypertension, heart condition, history of smoking, treated hypertension, systolic and diastolic blood pressure groups, BMI, and physical activity groups were entered into the backward elimination model as independent variables to create the final model shown in the table.

^b^ Diabetes is defined as self-report, Hba1c ≥6.5% or fasting glucose ≥126 mg/dL, or self-reported medication use)

^c^ Heart Condition is defined as self-reported coronary heart disease, angina, abnormal heart rhythm, heart attack, congestive heart failure or other heart condition

Using the alternative definition of PAD as an ABI <1.00, the prevalence was 9.6% (95% CI 8.5% to 10.7%). Multivariable logistic regression resulted in female sex (OR 1.81, 95% CI 1.30 to 2.50), diabetes (OR 1.80, 95% CI 1.34 to 2.42), less than high school education vs having a university degree (OR 1.70, 95% CI 1.07 to 2.69) and increasing systolic BP (OR 1.01, 95%CI 1.00 to 1.02) being statistically significant predictors of PAD (Table 4). If site was added to the model (Puerto Rico as the reference) then Barbados became an additional predictor (OR 1.57, 95% CI 1.09 to 2.28), and educational level was no longer significant.

**Table 4:** Unadjusted^a^ and adjusted odds ratios for predictors of Peripheral Arterial Disease (PAD) and Borderline PAD (ABI <1.00) compared to normal arteries (ABI 1.0 to 1.4).

Factor	Unadjusted Odds ratio (95% CI)	*P* value	Adjusted Odds ratio (95% CI)	*P* value
Female	2.08 (1.53 to 2.82)	<0.01	1.81 (1.30 to 2.50)	<0.001
**Education Level**				
Not completed High School	1.96 (1.28 to 3.00)	0.002	1.70 (1.07 to2.69)	0.024
Completed High School	1.79 (1.14 to 2.82)	0.012	1.50 (0.91 to 2.46)	0.111
Some college	1.70 (1.0 to 2.70)	0.402	1.62 (0.99 to 2.64)	0.054
University Degree	Reference	0.026	Reference	
Diabetes^b^	2.00 (1.51 to 2.64)	<0.001	1.80 (1.34 to 2.42)	<0.001
Systolic BP (mm Hg)	1.01 (1.01 to 1.02)	<0.001	1.01 (1.00 to 1.02)	0.012

^a^ Age, sex, education level, diabetes, hypertension, history of smoking, systolic and diastolic blood pressure, BMI, and physical activity groups were entered into the backward elimination model as independent variables.

^b^ Diabetes is defined as self-report, Hba1c ≥6.5% or fasting glucose ≥126 mg/dL, or self-reported of medication use

If PAD (ABI ≤0.9) and noncompressible arteries (ABI >1.4) were combined into one category then diabetes, having a heart condition and increasing BMI were predictors of this combined category (Table 5).

**Table 5:** Unadjusted^a^ and adjusted odds ratios for predictors of Peripheral Arterial Disease (ABI ≤0.90) compared to normal arteries (ABI 1.0 to 1.4) and non-compressible arteries (ABI >1.4) as a group.

Factor	Unadjusted Odds ratio (95% CI)	*P* value	Adjusted Odds ratio (95% CI)	*P* value
Diabetes^b^	1.82 (1.34 to 2.47)	<0.001	1.62 (1.18 to 2.21)	0.003
Heart condition	1.70 (1.21 to 2.40)	0.002	1.51 (1.04 to 2.19)	0.031
BMI (Kgm^-2^)	1.06 (1.04 to 1.08)	<0.001	1.06 (1.03 to 1.08)	<0.001

^a^ Age, sex, education level, diabetes, hypertension, heart condition, history of smoking, treated hypertension, systolic blood pressure, diastolic blood pressure, BMI, and physical activity groups were entered into the backward elimination model as independent variables.

^b^ Diabetes is defined as self-report, Hba1c ≥6.5 or fasting glucose >126 mg/dL, or self-reported medication use.

## Discussion

This study provides the first estimate of PAD prevalence in community-dwelling people 40 years of age and over resident in a multicultural group of Eastern Caribbean islands. The major findings were that for the cohort, 4.4% of participants had an ABI in the PAD range (ABI ≤0.9) in the leg evaluated. A further 5.2% had an ABI in the borderline range and 3.4% in the noncompressible range. Female sex, diabetes, a history of heart disease and low educational attainment were significant predictors of PAD. However, remarkably, age and cigarette smoking were not independently associated with PAD.

### Prevalence

PAD prevalence was estimated to be 4.3% in people 40 years of age and over by the 1999–2000 National Health and Nutrition Survey [1], and 3.6% in the Framingham offspring study [34]. Our prevalence of 4.4% is however not directly comparable since we measured the ABI in one leg only.

In a study of people with diabetes in Barbados, the unadjusted prevalence of PAD was 17.4% but when legs were considered individually 10.9% of legs had an ABI in the PAD range [27]. This means that many people with PAD in that study had only one leg affected, and assessing one leg only would miss PAD in 37% of cases. However, the San Diego population study found PAD was unilateral in 38% of cases [10] and testing one leg only would miss 19% of cases. On this basis the true prevalence of PAD per individual in our study may be between 5 and 7%.

BP taken with an oscillometric device may be lower than that done using doppler ultrasound. If an ABI cut-off of <1.0 was used [15, 21, 23] the prevalence of legs with PAD in our study would be 9.6%. The prevalence of individuals with PAD may be between 12 and 15%. Both the increased sensitivity and decreased specificity resulting from the use of a higher cutoff will contribute to the increase in estimated prevalence.

In our study women were more likely to have PAD than men (OR 1.72). A systematic review found that in HICs, women had a slightly higher PAD prevalence than men up to the age of 75 years. The prevalence was 7.8% in women vs 6.6% men at 55 to 59 years but it was 24.5% in women vs 27.4% in men at 85 to 89 years [9]. In LMICs there was little difference in prevalence between men and women [9]. The ECHORN cohort sites are all classified as high income economies by the World Bank [24].

### Factors associated with Peripheral Arterial Disease

Diabetes is a recognised risk factor for atherosclerotic disease including PAD. Many studies confirm this. Globally the odds ratio is 1.89 for people with diabetes with the risk being greater in HICs (OR 1.98) than in LMIC (OR 1.82) [9]. In our study the odds ratio was 2.23 which was similar to the NHANES study (OR 2.71) [1]. In a Barbados study the prevalence in a sample of newly diagnosed and known cases of diabetes was 18.6% with female sex and increasing age being independently associated with PAD [27].

Coronary artery disease and PAD are both clinical manifestations of atherosclerotic disease. It is therefore not surprising that a history of heart disease is associated with the presence of PAD. Studies in both lower and higher middle income countries have shown this association [9].

Education level is recognised as a major determinant of health disparity. Educational attainment is inversely associated with cardiovascular disease [35]. Low socioeconomic status is associated with an increased risk of cardiovascular disease [36].

The finding that people living in Barbados were more likely to have PAD than those from the other sites, suggests that factors not considered in the multivariable regression such as ethnicity may be important. Most people (92%) in Barbados are of African descent [37] whereas in Puerto Rico people of African descent are a minority [38].

### Factors not associated with Peripheral Arterial Disease

Guidelines suggest that people 65 years or age and over, or who are 50 to 64 years plus have risk factors are at increased risk of PAD [13, 39]. Prevalence rises sharply from age 70 [1, 11, 14] especially in HICs [9]. Unlike other studies [9] PAD was not independently associated with increasing age in our study, although in the bivariate analysis people with PAD were slightly older than those without PAD and there was a rise in prevalence at around age 70. There was little change in prevalence between 40 and 69 years of age and our study did not include people less than 40 years of age. Smoking, hypertension and hypercholesterolaemia, all recognised risk factors, were also not associated with PAD in our study. Unlike our study, in almost all other studies current smoking has been found to be one of the strongest risk factors for PAD, with current smoking vs non-smoking at least doubling the odds [9, 11]. While our study was larger than the 1999–2000 NHANES study which found smoking strongly associated with PAD, only about 8% of our study population as opposed to over 20% for NHANES were current smokers [1] and this would reduce the power to detect a difference. Using the alternative diagnosis for PAD of an ABI <1.00, increasing systolic BP was associated with PAD.

ABI readings above 1.4 indicate non-compressible arteries which in some cases may mask PAD [13]. We explored this by analysing PAD and noncompressible arteries as one group. Diabetes and having a heart condition remained predictors but female sex and educational level did not.

### Strengths and limitations

Representative population samples were recruited in 4 Eastern Caribbean islands and therefore, for each individual island, cohort members are representative of the general community dwelling population. However, men are underrepresented in the sample and cross-sectional studies, while being able to quantify associations, cannot determine causality. Some data were self-reported, and this may lead to inaccuracies especially with heart disease and physical activity.

This study used an oscillometric method with arm and ankle BP measured simultaneously and provides reproducible results with little inter-rater variability. However, oscillometry may yield slightly higher ABI readings than measurements with Doppler ultrasound [15, 21, 23]. Non-compressible arteries in some cases may mask PAD [13] and people with longstanding diabetes are more likely to fall into this category [13, 40]. One third of our study participants had diabetes. ABI is normally determined for each leg and the leg with the lower ABI is used to indicate whether PAD is present. We measured ABI in one leg only and this will not give the true population prevalence of PAD. The population prevalence will therefore be higher than our figures indicate.

## Conclusions

The PAD prevalence estimated by our study is higher than in comparable US studies. The estimate more than doubles if PAD is defined an ABI <1.0 as opposed to the traditional ≤0.9 recommended by guidelines [13, 41] and used in some epidemiological studies regardless of device used [9]. Since oscillometric devices are easy to use, less operator dependent and are becoming widely used, guidelines are needed to address the issue.

Our study confirms that diabetes is an important risk factor. It also finds that, in the Eastern Caribbean, women are at elevated risk of PAD and since most cases of PAD are asymptomatic it is important to screen women for this condition. However important differences with other studies are the lack of association of either increasing age or current cigarette smoking with PAD. The lack of association of cigarette smoking with PAD is an intriguing finding but the possibility of a type 2 error needs to be entertained. Low educational attainment was associated with PAD. PAD screening is not readily available in the countries studied putting people of lower socioeconomic status at a disadvantage.

Wave 2 of the ECHORN project will provide longitudinal data on PAD incidence and risk. Funding is being sought to bring cohort members back for a third follow up wave, at which time the ABI in both legs will be measured to provide a better PAD estimate. Future research should also seek to determine the factors that contribute to the significantly higher prevalence of PAD in Barbados compared to Puerto Rico.
